# Monoamine oxidase inhibition in cigarette smokers: From preclinical studies to tobacco product regulation

**DOI:** 10.3389/fnins.2022.886496

**Published:** 2022-08-16

**Authors:** Alan F. Sved, Jillian J. Weeks, Anthony A. Grace, Tracy T. Smith, Eric C. Donny

**Affiliations:** ^1^Departments of Neuroscience, Psychiatry and Psychology, University of Pittsburgh, Pittsburgh, PA, United States; ^2^Center for Neuroscience, University of Pittsburgh, Pittsburgh, PA, United States; ^3^Department of Psychiatry and Behavioral Sciences, Medical University of South Carolina, Charleston, SC, United States; ^4^Department of Physiology and Pharmacology, Wake Forest School of Medicine, Medical Center Boulevard, Winston-Salem, NC, United States

**Keywords:** nicotine, monoamine oxidase (MAO), cigarette addiction, tobacco, schizophrenia

## Abstract

Monoamine oxidase (MAO) activity is reduced in cigarette smokers and this may promote the reinforcing actions of nicotine, thereby enhancing the addictive properties of cigarettes. At present, it is unclear how cigarette smoking leads to MAO inhibition, but preclinical studies in rodents show that MAO inhibition increases nicotine self-administration, especially at low doses of nicotine. This effect of MAO inhibition develops slowly, likely due to plasticity of brain monoamine systems; studies relying on acute MAO inhibition are unlikely to replicate what happens with smoking. Given that MAO inhibition may reduce the threshold level at which nicotine becomes reinforcing, it is important to consider this in the context of very low nicotine content (VLNC) cigarettes and potential tobacco product regulation. It is also important to consider how this interaction between MAO inhibition and the reinforcing actions of nicotine may be modified in populations that are particularly vulnerable to nicotine dependence. In the context of these issues, we show that the MAO-inhibiting action of cigarette smoke extract (CSE) is similar in VLNC cigarettes and cigarettes with a standard nicotine content. In addition, we present evidence that in a rodent model of schizophrenia the effect of MAO inhibition to enhance nicotine self-administration is absent, and speculate how this may relate to brain serotonin systems. These issues are relevant to the MAO-inhibiting effect of cigarette smoking and its implications to tobacco product regulation.

## Cigarette smoking inhibits monoamine oxidase

Cigarette smoking is a serious health concern, with nicotine dependence contributing to premature disability and death due to the toxic properties of cigarette smoke. However, while it is clear that nicotine is the primary addictive component of cigarette smoke, the full nature of the addiction and the biological underpinnings are still a matter of debate as there are over 7,000 chemicals in cigarette smoke (Rodgman and Perfetti, 2013). Monoamine oxidase (MAO) activity is reduced in smokers compared to non-smokers (Berlin and Anthenelli, 2001; Fowler et al., 2003a; Hogg, 2016), and this may contribute to nicotine dependence in smokers. This review presents the evidence that the use of tobacco products, particularly the smoking of cigarettes, leads to inhibition of MAO and that this may contribute to the reinforcing properties of nicotine, thereby promoting the use of these tobacco products. While discussing the evidence in human tobacco users, we will focus on preclinical studies in experimental animals examining the potential mechanisms by which MAO inhibition may impact the reinforcing properties of nicotine and nicotine use.

Brain imaging studies document a decrease in brain MAO in chronic cigarette smokers, with a roughly 30–40% inhibition (Fowler et al., 2003a). Both isoforms of MAO are impacted, with inhibition of MAO-A being slightly greater than for MAO-B (Fowler et al., 1996a,b, 2003a; Leroy et al., 2009). MAO activity is also reduced in platelets and peripheral tissues in smokers compared to non-smokers and former smokers (Norman et al., 1987; Berlin et al., 1995b; Whitfield et al., 2000; Fowler et al., 2003b,^2005^; Launay et al., 2009). Furthermore, the extent of MAO inhibition, at least in platelets, is correlated with the amount of smoking, as reflected by blood cotinine or thiocyanate levels (Norman et al., 1982; Berlin et al., 2000). The time-course of decline in MAO activity following smoking cessation is measured in weeks (Rose et al., 2001; Fowler et al., 2003a; Gilbert et al., 2003) and suggests that substances in cigarette smoke cause an irreversible inhibition of MAO that persists until new enzyme is synthesized. Whereas a difference in MAO activity between smokers and non-smokers could result from either smoking causing a reduction in MAO activity or reduced MAO activity leading to increased likelihood of smoking, the evidence strongly supports the first possibility (Hogg, 2016).

Despite the evidence that cigarette smoking leads to a decrease in MAO activity, some studies suggest that low MAO activity may also promote smoking. An association between MAO gene polymorphisms consistent with lower MAO-A activity leading to an increased likelihood of smoking has been reported (McKinney et al., 2000; Jin et al., 2006; Tang et al., 2009; Tiili et al., 2017; Shen et al., 2019). In a series of longitudinal studies of smoking behavior in adolescents, Kiive et al. (2002); Harro et al. (2004), and Sakala et al. (2022) reported that low platelet MAO activity measured prior to the onset of smoking increased the likelihood of future smoking, as well as other drug use and risky behavior. However, this relationship between low MAO activity and increased likelihood of smoking has not been observed in all studies and a 2015 meta-analysis found that, if anything, the low activity alleles reduce the likelihood of heavy smoking (Yang et al., 2015), though not smoking overall. If low MAO activity increased smoking, it would be expected that the clinical use of MAO inhibitors may promote smoking, and this notion is not supported by the literature. Indeed, some researchers have suggested that MAO inhibitors may be useful smoking cessation treatments (Berlin et al., 1995a; Berlin et al., 2002; George and Weinberger, 2008), though this has not been supported by controlled studies of MAO-A or MAO-B inhibitors (Weinberger et al., 2010; Berlin et al., 2012; Kahn et al., 2012). This could be due to the difference between genetically driven low MAO throughout life, which would be expected to lead to compensatory changes particularly during the critical periods of development, that would not occur to a similar extent with MAO inhibitor treatment. Even so, as detailed below, preclinical studies in animal models suggest that MAO inhibition may promote nicotine self-administration. The preclinical studies suggest that the effect of MAO inhibition on nicotine self-administration is dependent upon nicotine dose, promoting self-administration of low doses of nicotine while reducing it with higher doses. Furthermore, the preclinical data provide evidence that the effect of MAO inhibition to promote nicotine self-administration might not be observed as an increase in cigarette smoking in at least some individuals with neuropsychiatric disorders, the population most likely to be treated with MAO inhibitors. These preclinical observations provide some rationale for the lack of evidence of MAO inhibitors increasing smoking.

## It is not clear what chemicals in cigarette smoke are responsible for the decrease in monoamine oxidase activity in smokers

Despite the MAO-inhibiting effect of cigarette smoking being known for more than 25 years, the mechanism by which this occurs is still not clear. Several chemicals in cigarette smoke have been identified that inhibit MAO (Castagnoli et al., 2002; Lewis et al., 2007; Hogg, 2016), but it seems that none of them are present in high enough concentrations to produce the observed inhibition of MAO. Harmane, norharmane, and 2-naphthylamine have been shown to be present in cigarette smoke and inhibit MAO (Khalil et al., 2000; Hauptmann and Shih, 2001; Rommelspacher et al., 2002; Herraiz, 2004; Khalil et al., 2006), but the evidence that these compounds, either individually or collectively, are responsible for the MAO inhibition observed in cigarette smokers is unconvincing (Hogg, 2016). Numerous studies have shown that cigarette smoke extracts (CSE) display MAO-inhibiting activity in *in vitro* assays, but it is unclear whether the identified MAO inhibitors in smoke can account for this. Maybe all of the relevant MAO-inhibiting substances in cigarette smoke have not yet been identified, possibly because they are not captured in smoke extracts, or maybe the known MAO inhibitors interact in a more than additive manner to inhibit MAO. As discussed below, it is also possible that these studies have not been of a sufficiently chronic nature to allow the effect to develop. The possibility must also be considered that MAO inhibition in cigarette smokers is an effect of metabolic products of the constituents of cigarette smoke or the production of some endogenous MAO inhibitor caused by smoking, and therefore MAO inhibition in cigarette smokers will not be fully explained by studying smoke extracts.

Whereas smoking commercially available cigarettes causes MAO inhibition, it is important to consider whether this is an effect produced by all tobacco products or even all combustible tobacco products. Smoke extract from “roll your own” cigarettes produced MAO inhibition in *in vitro* assays (Lewis et al., 2012; Truman et al., 2017), consistent with MAO inhibition being an intrinsic property of combusted tobacco rather than something about the manufacture of commercial cigarettes. Indeed, the extent of MAO-inhibiting activity of smoke extracts for “roll your own” cigarettes exceeded that of extracts from commercial cigarettes (Lewis et al., 2012), though it is not clear what chemicals are responsible for this difference (Truman et al., 2017).

**FIGURE 1:**
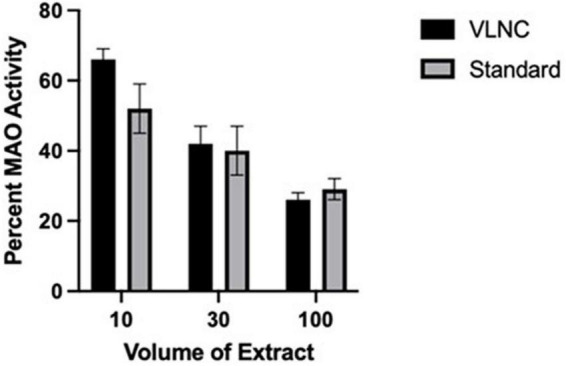
MAO inhibition by Spectrum VLNC cigarette extract. Aqueous cigarette smoke extract was produced using Spectrum cigarettes nicotine content of 0.4 and 15.8 mg nicotine per gram of tobacco, using a method modified from Gellner et al. (2016a,b). Extracts were prepared from individual cigarettes by bubbling smoke through a 10 ml column of water 8 cm high in 15 ml conical bottom test tubes. 40 ml of smoke was drawn into a syringe during 1.5–2.0 s and then pushed out of the syringe through the column of water through a 22 ga stainless steel tube during 20–25 s. The 40 ml “breaths” of smoke were performed every 30 s, for 14–15 breaths per cigarette. Extract and dilutions of extract in saline were tested for MAO inhibition using recombinant human MAO-A (Sigma Chemicals). MAO activity was assayed as previously described (Smith et al., 2016), using 2 μg of MAO-A per assay well for the MAO activity. Each extract dilution was assayed in duplicate and 6 separate extracts were prepared from each cigarette type. Extract was assayed for MAO-A-inhibiting activity by adding 10–100 μl of extract to the assay well (out of a total of 200 μl volume). There was a statistically significant effect (*p* < 0.01) of extract volume (with each increasing volume producing a greater inhibition of MAO activity) with no effect of extract type and no interaction.

Given that the United States Food and Drug Administration is formally considering a mandated reduction in the nicotine content of combustible tobacco (Gottlieb and Zeller, 2017; Hatsukami et al., 2022), and similar approaches to reducing the impact of cigarettes on public health are being considered in other countries with New Zealand leading the way (Smokefree Aotearoa 2025, 2021), it is important to know whether very low nicotine content (VLNC) cigarettes have a similar effect on MAO compared to standard cigarettes. To the extent that MAO inhibitors are found in tobacco and are aerosolized upon combustion, it would be expected that smoke from VLNC cigarettes would show MAO-inhibiting activity similar to standard cigarettes. To test this hypothesis, we compared CSE s from Spectrum cigarettes with a standard nicotine content (15.8 mg/g tobacco) and a VLNC (0.4 mg/g tobacco). As illustrated in Figure 1, they share very similar MAO-inhibiting activities assayed *in vitro*.

MAO-inhibiting activity is not just found in smoke from combusted tobacco, it is also present in tobacco leaf extracts (Khalil et al., 2000; Castagnoli et al., 2002) and smokeless tobacco products (e.g., SNUS) (Van Der Toorn et al., 2019). However, the MAO inhibitors do not appear to be aerosolized by moderate heating of tobacco, as aerosols derived from “heat not burn” products do not appear to inhibit MAO in *in vitro* assays (Van Der Toorn et al., 2019). Whereas most e-cigarette vaping solutions do not appear to inhibit MAO (Truman et al., 2019; Van Der Toorn et al., 2019) some flavored vaping solutions do contain MAO-inhibiting compounds (Truman et al., 2019). To date, the flavorants found to have significant MAO-inhibiting activity are vanillin and related compounds (found in vanilla bean flavored vaping solutions) (Truman et al., 2019), but that does not mean that other chemicals with MAO-inhibiting activity will not be found as more e-liquids get tested. Thus, MAO-inhibiting activity needs to be considered for a variety of tobacco and smoking-related products.

## Monoamine oxidase inhibition promotes nicotine self-administration in preclinical studies

**FIGURE 2:**
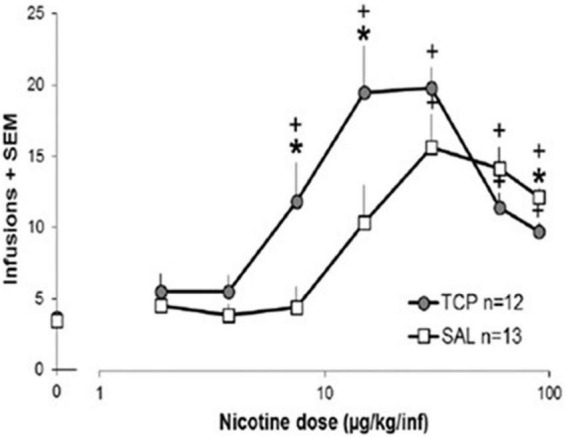
Effect of MAO inhibition on nicotine self-administration in rats. Rats were treated with tranylcypromine (TCP, 1 mg/kg ip) or saline vehicle 1 h prior to the start of each daily 1-h nicotine self-administration sessions. Rats were tested with ascending doses of nicotine, with the dose increasing every 7 days. Data were analyzed as the average of the final 3 days on each dose for each rat (for details, see Smith et al., 2016, and this figure is reproduced from that publication). A significant difference (*p* < 0.05) between the TCP group and the SAL group at a single dose is represented by * and a significant difference from 0.0 ug/kg/infusion is represented by +.

In contrast to the unclear picture provided by clinical studies, studies in rats demonstrate that MAO inhibition promotes nicotine self-administration (Guillem et al., 2005; Guillem et al., 2006; Villegier et al., 2007; Smith et al., 2015, 2016). While there are many caveats in translating findings from nicotine self-administration in rats to smoking in people, nicotine self-administration studies have often provided useful insight (Donny et al., 2012; Smith et al., 2017). Treatment of rats with drugs that inhibit MAO increases nicotine self-administration, primarily by shifting the dose-response curve for nicotine to the left (Figure 2). Importantly, with MAO inhibition the threshold dose of nicotine supporting self-administration is significantly decreased (Guillem et al., 2005; Villegier et al., 2007; Smith et al., 2016). This effect of MAO inhibition to increase nicotine self-administration is observed on a variety of fixed ratio schedules of reinforcement as well as with progressive ratio schedules (Guillem et al., 2005; Smith et al., 2016). MAO inhibition also increases the effect of nicotine on enhancing responding for other reinforcing stimuli (the reinforcement enhancing effect of nicotine) (Smith et al., 2016), which may play a substantial role in the overall reinforcing effects of nicotine (Caggiula et al., 2009; Rupprecht et al., 2015). The effect of MAO inhibition on shifting the dose-response curve for nicotine self-administration also pertains to the descending limb of the inverted-U-shaped curve, with this dose-related decrease in self-administration that is typically seen at high doses of nicotine seen at more moderate doses in MAO-inhibited rats (Guillem et al., 2005; Smith et al., 2016). This shifting of the dose response curve suggests that MAO inhibition is not selectively impacting either the reinforcing or rate-limiting (e.g., aversive) effects of nicotine. Interestingly, because of the leftward shift of the inverted-U-shaped nicotine dose-response curve produced by MAO inhibition, the effect of MAO inhibition is minimal at moderate doses of nicotine, doses that are often used in nicotine self-administration studies. This may also explain why MAO inhibitors used as clinical treatments have not been reported to impact smoking. Furthermore, at high doses of nicotine MAO inhibition has the opposite effect on nicotine self-administration and this may also pertain to heavy smokers.

The effect of MAO inhibition in promoting nicotine self-administration in rats results from inhibition of MAO-A, as selective MAO-A inhibitors show the same effect as non-selective MAO inhibitors, whereas selective MAO-B inhibitors are typically without effect (Guillem et al., 2006; Smith et al., 2016). Most preclinical studies examining the effect of MAO inhibition on nicotine self-administration have used very large doses of MAO inhibitors that would be expected to produce extensive inhibition of MAO and, as discussed below, produce off-target effects that likely impact the interpretation of the results. Importantly, an effect on nicotine self-administration is still observed using smaller doses of MAO inhibitors to mimic the ∼40% inhibition of MAO observed in cigarette smokers (Fowler et al., 2003a), an effect on nicotine self-administration is still observed (Smith et al., 2016).

## Mechanism by which monoamine oxidase inhibition increases nicotine self-administration

There is some confusion in the literature regarding the mechanism by which MAO inhibitors, such as tranylcypromine, increase nicotine self-administration. For example, Lotfipour et al. (2011) studied the effect of large doses of tranylcypromine (3 mg/kg) administered daily for 4 days and concluded that the effect of this drug on increasing nicotine self-administration was not simply a result of MAO inhibition, since 20 h after injection of tranylcypromine MAO was still inhibited but nicotine self-administration was not altered. Villegier et al. (2011) provided additional evidence that this large dose of tranylcypromine increased low dose nicotine self-administration by a combined action of MAO inhibition and increased serotonin release. A key issue here seems to be the time course of the effect and the use of a large dose of tranylcypromine, a dose that causes serotonin release as well as inhibiting MAO (Baker et al., 1992; Villegier et al., 2007). As discussed below, inhibiting MAO activity for just a few days is not sufficient to see the effect of MAO inhibition on nicotine self-administration; rather, MAO inhibition needs to be more chronic, persisting for a week or more (Smith et al., 2015, 2016), which means that the effect of MAO inhibition of nicotine self-administration is not a direct effect of MAO inhibition *per se*, but additionally requires some chronic adaptation to MAO inhibition. Such differences are highlighted by comparing the time course to the effects observed by, for example, Lotfipour et al. (2011) and Villegier et al. (2011) with those of Smith et al. (2015, 2016). Clearly, the mechanisms underlying the acute effects of large doses of tranylcypromine are different from the more chronic effects of lower doses of this drug, which are likely the result of chronic MAO inhibition. Two comparisons between these sets of studies are revealing. Whereas Lotfipour et al. (2011) and Villegier et al. (2011) report that 20 h after 4 daily doses of a large dose of tranylcypromine (3 mg/kg) nicotine self-administration is not increased though MAO is still inhibited, Smith et al. (2016) show that a week or more after a smaller dose of tranylcypromine (e.g., 1 mg/kg) nicotine self-administration is increased to the same extent whether tested 1 h or 20 h after dosing with the MAO inhibitor. This difference in time course is consistent with the development of changes in receptor sensitivity. Also, whereas Villegier et al. (2011) show that doses smaller of tranylcypromine (<3 mg/kg) do not impact nicotine self-administration when tested for up to 4 days, Smith et al. (2016) report an effect of tranylcypromine doses as low as 0.1 mg/kg when the drug is administered for more than 2 weeks. Nonetheless, it is important to note that acute (and up through at least 5 days) MAO inhibition alone is not sufficient to impact nicotine self-administration. Rather, this effect of MAO inhibition on nicotine self-administration is an effect that develops over many days, and therefore likely involves adaptations to the MAO-inhibited state. Indeed, there is some evidence as to what the key adaptation may be. Lotfipour et al. (2011) and Villegier et al. (2011) demonstrated that during the first several days of MAO inhibition produced by a large dose of tranylcypromine there is no enhancement of nicotine self-administration unless it is also accompanied by increased serotonin release, either as a direct effect of this large dose of tranylcypromine or produced by a different serotonin-releasing drug. While MAO inhibition would be expected to increase synaptic serotonin by inhibiting its metabolism, this would be opposed by stimulation of 5HT1a autoreceptors (Artigas, 2013). However, over the course of days, these 5HT1a autoreceptors desensitize, allowing MAO inhibition to increase synaptic serotonin levels. Indeed, this mechanism is hypothesized to underlie the delayed onset of many antidepressant drugs, including MAO inhibitors, tricyclics, and selective serotonin reuptake inhibitors (SSRI) (Gartside et al., 1997; Sharp et al., 1997; Cryan and Leonard, 2000; Hjorth et al., 2000; Artigas, 2013). Such a scenario makes the apparently conflicting data with MAO inhibitors merge into a consistent framework in which MAO inhibition increases nicotine self-administration through a mechanism including serotonergic signaling. Another point that emerges from this discussion is that in order to study the potential effect of a putative MAO-inhibiting substance in tobacco products, the substance needs to be tested over a prolonged timeframe.

## Monoamine oxidase inhibition by cigarette smoke and nicotine reinforcement

These studies showing an effect of MAO inhibition enhancing nicotine self-administration mostly relied on MAO-inhibiting drugs (e.g., TCP, clorgyline) that are not present in cigarette smoke. Arnold et al. (2014) reported that norharmane (2.5 μg/kg/inf) was self-administered by rats and this was additive with nicotine (7.5 μg/kg/inf). However, the dose of norharmane used in that study is roughly 10-fold higher than what might be expected from cigarette smoke given its concentration relative to nicotine (Herraiz, 2004) and there is also no evidence that this dose resulted in inhibition of MAO. Guillem et al. (2006) and Hall et al. (2014) examined the effect of large doses of norharmane on nicotine self-administration; Hall et al. (2014) found an increase in nicotine self-administration produced by acute treatment with norharmane whereas Guillem et al. (2006) reported no effect with chronic norharmane treatment. Smith et al. (2015) reported that a cocktail of cigarette smoke constituents, at doses expected to be in the range expected to be in cigarette smoke, including harmane and norharmane, did not impact nicotine self-administration using a variety of nicotine doses and schedules of reinforcement (tranylcypromine did increase nicotine self-administration in that study). Importantly, there is no evidence that the doses of harmane or norharmane used in any of those studies were large enough to produce significant inhibition of MAO *in vivo*.

If CSE inhibits MAO and MAO inhibition promotes nicotine self-administration (particularly at low doses of nicotine), then CSE should support self-administration to an extent greater than nicotine alone. Indeed, some studies have shown that CSE produces a greater effect on nicotine self-administration than can be explained based solely on nicotine content, though none of these studies document *in vivo* MAO inhibition by CSE. Possibly the most compelling differences between self-administration of nicotine and CSE is reported by Costello et al. (2014), though this response was not replicated in subsequent studies (Gellner et al., 2016a; Cross et al., 2020; possibly because of small methodological details, Gellner et al., 2016a). Costello et al. (2014) reported that an aqueous extract of cigarette smoke increased self-administration of smaller doses of nicotine compared to nicotine alone, a shift of the nicotine dose-response curve consistent with the effects of MAO inhibition noted above; similar results were reported by Marusich et al. (2019). While the CSE used by Costello et al. (2014) inhibited MAO activity assayed *in vitro*, evidence of *in vivo* MAO inhibition was not provided. Furthermore, the impact of the increased self-administration of CSE compared to nicotine alone appeared to occur more rapidly than what has been observed with MAO-inhibition. Also, compared to nicotine alone, CSE did not increase responding for nicotine on a progressive ratio schedule of reinforcement, in contrast to what has been reported to MAO inhibition. Brennan et al. (2015) reported that an ethanolic extract of tobacco smoke particulate matter from “roll your own” cigarettes produced greater self-administration than nicotine alone, but this was not observed with extract from commercial cigarettes; interestingly, extracts from “roll your own” cigarettes also display greater MAO-inhibiting activity compared to commercial cigarettes (Lewis et al., 2012). Levin et al. (2021), using a similarly generated extract from Kentucky Research Cigarettes, also reported that the extract did not produce greater self-administration compared to nicotine alone. Several of these studies of CSE measured the harmane and norharmane concentrations of the extract (Costello et al., 2014; Brennan et al., 2015; Marusich et al., 2019) and the concentrations in the self-administration solutions are more than 10-fold lower than needed to inhibit MAO (Herraiz and Chaparro, 2005). Thus, even if CSE does provide for greater self-administration than nicotine alone, a conclusion that is not consistently supported by the published data, there is no evidence at present that this effect of CSE might be caused by MAO inhibition. However, there are several potential reasons why this conclusion might not be accurate. The effect of MAO inhibition on nicotine self-administration is most apparent at low doses of nicotine and in order to test these low doses in CSE the whole extract needs to be diluted, thereby potentially reducing the impact of MAO-inhibiting compounds. This could be tested using CSE produced from VLNC cigarettes that then gets supplemented with nicotine. Also, although the impact of MAO inhibition on nicotine self-administration may take 2 weeks to develop, many experiments with CSE do not follow such a prolonged time course. Furthermore, without knowing the nature of the chemicals in cigarette smoke that lead to MAO inhibition, it is possible that they are not adequately extracted or stable using the current methods.

## Other preclinical evidence of an interaction between nicotine and monoamine oxidase inhibition on reinforcement

Intracranial self-stimulation (ICSS) can be used as another approach to examine the interaction between nicotine and MAO inhibition on reinforcement (Negus and Miller, 2014). In this paradigm, rats respond on a lever to receive electrical stimulation of brain reward pathways and other reinforcing stimuli reduce the electrical threshold required to elicit ICSS. Harman and norharmane, even in large doses that would be expected to inhibit MAO, did not reduce the threshold for nicotine ICSS and may even increase it (Harris et al., 2020), though this was tested only in acute experiments. To our knowledge, other MAO inhibitors (e.g., tranylcypromine, clorgyline) and more long-term MAO inhibition have not been tested in that paradigm. Similarly, it does not appear that MAO inhibitors have been tested on nicotine reinforcement in other behavioral tests of reinforcement, such as conditioned place preference (CPP). We hypothesize that chronic MAO inhibition would enhance reinforcement by low doses of nicotine in both paradigms.

The results of studies on the effects of MAO inhibitors on nicotine self-administration in rodents lead to the hypothesis that mice in which the MAO-A gene has been deleted should show enhanced nicotine reinforcement at low doses of nicotine. While no studies reported to date have examined the effect of reduced MAO-A gene expression on nicotine self-administration, one study (Agatsuma et al., 2006) has examined nicotine-induced CPP in MAO-A knockout mice. Interestingly, that study did not observe an enhanced nicotine-induced CPP, but rather reports a small reduction in this test of nicotine reinforcement. While it is unclear why these results seem to differ from the effect of MAO-inhibition on nicotine self-administration behavior, this could reflect differences in brain development in the absence of MAO-A, especially during critical period where effects opposite to inhibition during adulthood might occur. Unfortunately, it is difficult to compare studies that differ in species, behavioral assay, and mechanism of MAO reduction, so more research is required to understand these differences.

## Interaction between monoamine oxidase inhibition and other behavioral actions of nicotine

Several studies have found that MAO inhibition with tranylcypromine or other MAO inhibitors increased nicotine-evoked locomotor activity in rats and mice (Villegier et al., 2006, 2010; Lanteri et al., 2009). Interestingly, this effect appears to involve MAO-inhibition induced desensitization of 5-HT1A receptors (Lanteri et al., 2009), as suggested above for MAO-inhibition evoked increases in nicotine self-administration. There are also reports of MAO inhibition enhancing the discriminative stimulus properties of nicotine in adult rats (Wooters and Bardo, 2007) and MAO-inhibition plus nicotine producing antidepressant-like actions in the forced swim test in adolescent, but not adult, rats (Villegier et al., 2010). These studies were conducted using acute dosing with MAO inhibitors, so it would be useful to determine how this might differ with a more prolonged timecourse of MAO inhibition, such as that required to enhance nicotine self-administration.

Of particular relevance to smoking dependence, Malin et al. (2013) reported that acute MAO inhibition in rats by injection clorgyline and deprenyl increased somatic signs of nicotine withdrawal, an effect mimicked by selective MAO-A inhibition. It would be interesting to see the impact of nicotine withdrawal in rats treated chronically with nicotine and MAO inhibition, across a range of nicotine doses.

## Is the effect of monoamine oxidase inhibition on nicotine self-administration potentially altered in smoking-vulnerable populations?

Individuals with neuropsychiatric disorders or chronic pain have a greater incidence of smoking than the general population (Lawrence et al., 2009; McClave et al., 2010; Larowe and Ditre, 2020) raising the question of whether MAO-inhibition produced by smoking cigarettes may be relevant to smoking in these particular populations. For example, individuals with depression smoke cigarettes at a higher rate than the general population (Klungsoyr et al., 2006) and pharmacologic MAO inhibition has antidepressant effects (Finberg and Rabey, 2016). Unfortunately, little work has been done to examine the role of MAO inhibition in smoking among individuals with depression and so at present it is unclear whether MAO inhibition plays any role in smoking in individuals with depression (or in any other population of increased vulnerability to smoking). A recent study in a rodent model of depression, the Flinders Sensitive Line (FSL) rat strain (Overstreet and Wegener, 2013), reported that nicotine may have a greater reinforcing efficacy in these rats (Smethells et al., 2021). Thus, this model may be an excellent one to test the interaction between nicotine and MAO-inhibition in depression.

**FIGURE 3:**
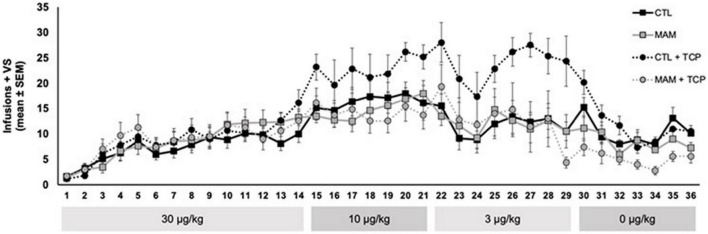
Effect of MAO inhibition on nicotine self-administration in MAM vs. control (CTL) rats. Adult MAM and CTL rats were injected with TCP (1.0 mg/kg, i.p.) or vehicle 1 h prior to behavioral sessions. Each group was 7–8 rats and included approximately equal numbers of males and females. Rats were allowed to self-administer nicotine (30 μg/kg, i.v.) paired with a mildly reinforcing visual stimulus (VS) in daily 1-h behavioral sessions 5 days per week for 14 sessions. Descending doses of nicotine (10, 3, and 0 μg/kg) were self-administered for 7–8 sessions and catheter patency was confirmed at the end of each dose phase (for details related to the MAM rats and the self-administration protocol, see Weeks et al., 2020). (This study was approved by the University of Pittsburgh Animal Care and Use Committee and were conducted in accordance with the NIH Guide for the Care and Use of Laboratory Animals).

Individuals with schizophrenia also smoke at a much higher rate than the overall population (Dickerson et al., 2018). During the course of studying the effects of nicotine in a rodent model of schizophrenia (Weeks et al., 2020, 2021) to help understand the high incidence of smoking in individuals with schizophrenia, we conducted an experiment to examine the impact of MAO inhibition on nicotine self-administration in this model. Utilizing a developmental model of schizophrenia in rats produced by injection of methylazoxymethanol acetate (MAM) into pregnant rats on day 17 of gestation (Moore et al., 2006), which is possibly the best available rodent model of schizophrenia (Modinos et al., 2015), we had observed that there was no change in nicotine self-administration among MAM offspring compared to control offspring (Weeks et al., 2020). We went on to address the hypothesis that an exaggerated interaction between MAO inhibition and nicotine might occur in MAM rats. In this experiment groups of MAM rats and control rats received intraperitoneal injections of the MAO inhibitor tranylcypromine (1.0 mg/kg ip) or saline vehicle 1 h before daily 1-h nicotine self-administration sessions. As shown in Figure 3, control rats displayed the expected MAO inhibition induced shift of the nicotine dose-response curve and, as in our prior observations, nicotine self-administration did not differ between MAM rats and control rats. However, in contrast to our hypothesis, the effect of MAO inhibition on nicotine self-administration was absent in the MAM rats. Thus, unlike control rats, in which MAO inhibition increased nicotine self-administration at low doses of nicotine, in MAM rats MAO inhibition had no effect on nicotine self-administration. These data illustrate that, to the extent that the MAM model in rodents replicates what occurs in individuals with schizophrenia, an interaction between MAO inhibition and the reinforcing effects of nicotine is not the explanation for increased smoking in individuals with schizophrenia. Another important implication of these data in MAM rats is that a potential interaction between MAO inhibition and nicotine reinforcement may not occur across all populations of smokers.

These data may also help to provide some insight as to the mechanism by which MAO inhibition increases nicotine self-administration, as it appears to be disrupted in the MAM rats. MAO inhibition would be expected to increase baseline extracellular levels of monoamines, particularly dopamine and serotonin with MAO-A inhibition in rats. Given the role of dopamine in drug self-administration, including the self-administration of nicotine, it is tempting to speculate that increased dopaminergic tone could result in a shift of the nicotine dose-response curve. While this could explain a potentiation of low-dose nicotine self-administration, it is harder to explain why increased dopamine tone would also shift the descending limb of the dose-response curve at higher doses. Additionally, as the MAM model of schizophrenia is thought to have elevated dopaminergic tone in the baseline state, it would be difficult to explain why MAO inhibition does not impact nicotine self-administration in this model (and also why baseline nicotine self-administration is not different). This complexity may involve heterogeneity of brain dopamine systems, with different DA systems mediating the ascending and descending limbs of the dose-response curve. For example, the heterogenous population of dopamine neurons in the ventral tegmental area with different inputs and projection fields are differentially parts of circuits involved with reward or aversion (Ikemoto, 2007; Lammel et al., 2014). As noted above, serotonergic systems may be involved in the effect of MAO inhibition on enhancing nicotine self-administration. Whereas it is possible that elevated serotonergic tone in response to chronic MAO inhibition could be responsible for the shift in nicotine self-administration, at present it is difficult to explain why this would be absent in the MAM-treated rats, unless there is some change in serotonergic systems in these rats. Unfortunately, serotonin systems have not been examined in this rodent model of schizophrenia or other similar models. Still, there is evidence for disruption in serotonergic systems in schizophrenia (Breier, 1995). Furthermore, disruption of serotonergic input to the dorsal hippocampus, an area impacted in the MAM model (Moore et al., 2006; Modinos et al., 2015), increases phencyclidine-induced hyperactivity and disrupts prepulse inhibition of startle (Adams et al., 2008), which are schizophrenia-like responses also observed in MAM rats (Modinos et al., 2015). These observations lead to testable hypotheses for why MAO inhibition fails to alter nicotine self-administration in the MAM model of schizophrenia.

## Considerations for tobacco regulatory policy

Cigarette smoking causes inhibition of MAO and, based on preclinical studies, MAO inhibition causes an increase in nicotine self-administration at low doses of nicotine. Nicotine and tobacco regulatory policy needs to weigh all factors promoting the use of nicotine and tobacco products, and MAO inhibition appears to be one of those factors requiring consideration. With current nicotine regulatory policy focusing on the potential of reducing nicotine levels in cigarettes to below an addictive level (FDA Tobacco Product Standard for Nicotine Level of Combusted Cigarettes), one of the critical questions for regulating nicotine and tobacco products pertains to the threshold dose at which nicotine becomes addictive. In this context, the preclinical observation that MAO inhibition significantly reduces the threshold dose supporting nicotine self-administration in rodents under specific conditions cannot be ignored. Setting allowable nicotine levels to the lowest possible level may reduce the likelihood that MAO inhibition could maintain nicotine reinforcement and continued use. As current clinical research has used VLNC cigarettes that propose inhibition of MAO similar to what occurs in standard cigarettes, trials of VLNC cigarettes should already account for MAO inhibition. However, monitoring MAO- inhibiting activity of new products would ensure against significant increases in MAO-inhibiting activity that might further alter sensitivity to low doses of nicotine do not occur. Further, non-cigarette tobacco products cannot be assumed to have the same threshold for nicotine reinforcement given that the level of MAO inhibition produced differs across products. Additionally, an interaction between MAO inhibition and the use of nicotine and tobacco products may vary substantially across different sub-populations of smokers; surprisingly, the impact of MAO inhibition on nicotine self-administration was absent in a rodent model of schizophrenia. Although this observation argues against MAO inhibition promoting nicotine use in individuals with schizophrenia, it does highlight the point that the relationship between MAO inhibition and tobacco use is not constant across populations and needs to be addressed in subpopulations with particular vulnerability to nicotine use, such as individuals with depression or chronic pain, and this could be considered in the context of regulatory policy.

## Summary and Conclusion

Cigarette smoking results in inhibition of brain MAO activity, though it is currently unclear which elements in cigarette smoke account for this MAO inhibition and how it generalizes to other tobacco products. While there is no direct evidence that MAO inhibition contributes to tobacco dependence in human smokers, experiments utilizing nicotine self-administration in rodents document that chronic MAO-inhibition increases self-administration of low doses of nicotine. The mechanism by which MAO inhibition promotes self-administration of nicotine in rodents is not yet clear, but chronic adaptations of serotonergic systems may be involved. Given that certain populations may be particularly vulnerable to smoking and tobacco dependence, e.g., individuals with depression, schizophrenia, or chronic pain, it is possible that smoking-induced MAO inhibition may contribute to the high incidence of smoking in these populations. Tobacco regulatory policy needs to consider how nicotine interacts with other chemicals in tobacco products, including the potential for MAO inhibition produced by cigarette smoke, to moderate the public health impact of potential policies.

## Author contributions

JW and AS conducted the experiments presented in the manuscript. All authors contributed to the development and writing of the manuscript.
